# Phytochemical Profiling of Methanolic Fruit Extract of *Gardenia latifolia* Ait. by LC-MS/MS Analysis and Evaluation of Its Antioxidant and Antimicrobial Activity

**DOI:** 10.3390/plants10030545

**Published:** 2021-03-13

**Authors:** Y. Mohan Reddy, S. P. Jeevan Kumar, K. V. Saritha, P. Gopal, T. Madhusudana Reddy, Jesus Simal-Gandara

**Affiliations:** 1 Department of Biotechnology, Sri Venkateswara University, Tirupati 517502, A.P., India; mohanbioteck@gmail.com; 2 Seed Biotechnology Laboratory, ICAR-Indian Institute of Seed Science, Mau 275103, Uttar Pradesh, India; jeevanicar@gmail.com; 3 ICAR-Directorate of Floricultural Research, Pune 411005, Maharashtra, India; 4 Electrochemical Research Laboratory, Department of Chemistry, Sri Venkateswara University, Tirupati 517502, A.P., India; pgopal1802@gmail.com (P.G.); tmsreddysvu@gmail.com (T.M.R.); 5 Nutrition and Bromatology Group, Department of Analytical and Food Chemistry, Faculty of Food Science and Technology, Ourense Campus, University of Vigo, E32004 Ourense, Spain

**Keywords:** antimicrobial activity, antioxidant activity, differential pulse voltammetry, *G. latifolia*, LC-MS/MS analysis

## Abstract

*Gardenia latifolia* Ait. (Rubiaceae) is also known as Indian Boxwood is a small deciduous tree often growing in southern states of India. In the present study, phytochemical profiling of methanolic extract of *G. latifolia* fruits were carried out using FTIR and LC-MS/MS analysis. Besides, its antioxidant and antimicrobial potential have been analysed using DPPH activity, differential pulse voltammetry and resazurin microtiter assay, respectively. Phytochemical profiling revealed the presence of 22 major diversified compounds and main were 3-caffeoyl quinic acid (chlorogenic acid), 3,4-Di-O-caffeoyl quinic acid, 6-O-trans-feruloylgenipin gentiobioside, 10-(6-O-trans sinapoyl glucopyranosyl) gardendiol, isoquercitrin, scortechinones, secaubryenol, iridoids and quercetin 3-rutinoside (rutin). The extract showed antioxidant activity (IC_50_ = 65.82) and powerful antibacterial activity with lowest minimum inhibitory concentration against Gram-positive *Staphylococcus aureus* (15.62 µg/µL), *Bacillus subtilis* (31.25 µg/µL) than gram negative *Escherichia coli* (62.5 µg/µL), *Klebsiella pneumoniae* (62.5 µg/µL), *Pseudomonas aeruginosa* (31.25 µg/µL). This study shows that the fruits of *G. latifolia* have tremendous potential to be used in food industries, phyto-therapeutics and cosmetic industries.

## 1. Introduction

*Gardenia latifolia* Ait. (Indian Boxwood) is a tree species belongs to the family Rubiaceae, which is habituated in southern parts of India particularly in dry forests. It has been used not only as a toy making species but also has medicinal properties owing to the presence of secondary metabolites. *G*. *latifolia* fruits are reported to be used as folk medicine for treating various ailments such as haemorrhage in humans, skin diseases, dental caries, stomach pain, snake bite and ephemeral fever in live stocks [1,2,3]. Besides, the fruit extract is also used as dye and food additives due to high content of yellow pigments [4]. *G*. *latifolia* fruit is a globose berry having crowned calyx and fruits round the year with rugose seeds. *G*. *latifolia* fruit is rich in nutrients and medicinal properties but detailed phytochemical analysis responsible for these medicinal properties has been poorly explored. 

Some attempts have been made to elucidate the phytochemical compounds and their potential as antioxidants, antimicrobial, anti-diabetic and anti-inflammatory compounds [5,6]. Reddy et al. [7] identified some phyto-constituents in stem bark such as hederagenin, D-mannitol, sitosterol and siaresinolic, episiaresinolic, oleanolic and spinosic acids. However, detailed exploration of phytochemical compounds in fruit extract of *G. latifolia* is limited [5,8,9]. Therefore, owing to higher efficiency and solubility of compounds in methanol, delineation of compounds in methanolic fruit extract of *G. latifolia* is quintessential. Determination of antioxidant activity is generally done by various techniques such as photometric, luminescence measurements, fluorimetric, HPLC, thermogravimetric, MS and GC but these procedures confront several drawbacks such as usage of specific reagents, requirement of exorbitant equipment and take much time and volume in sample preparation [10]. Similarly, to screen natural products for antimicrobial property deployment of reliable, efficient, rapid and cost-effective method is inevitable. Besides, quantity of natural products is meagre and is become a limiting factor for any screening programme [11,12]. Conventionally, disc diffusion method was employed to screen the natural products against antimicrobial activity, but it is time-consuming, require significant quantities of test materials [13]. Thus, efficient methods for screening of antioxidant and antimicrobial methods from natural products is essential. Hence to address these issues, in this study methanolic extract of *G. latifolia* was analysed by FT-IR and LC-MS/MS. Thereafter, antioxidant activity and antimicrobial properties have been evaluated with differential pulse voltammetry and resazurin assay, which are easy, rapid, and reliable and require very low amount of testing volume and time.

## 2. Results and Discussion

### 2.1. Phytochemical Screening of G. latifolia Fruit Extract

Selection of solvent systems for phytochemical extraction from *G. latifolia* has been done using hexane, chloroform, ethyl acetate and methanol. Among various solvents evaluated in the study, methanolic extract showed presence of alkaloids, saponins, glycosides, flavonoids and particularly phenols and terpenoids. In hexane, no compounds were present, while chloroform manifested the presence of phenols and flavonoids. Ethyl acetate showed presence of phenols, flavonoids, glycosides and terpenoids (Table S1). Presence of majority compounds in methanolic fruit extract implies that the solvent is having potential owing to its higher efficiency and solubility of phytochemical compounds. Hence, characterization of phytochemical compounds from *G. latifolia* has been done using methanol. Phenolic compounds are important class of secondary metabolites in plants that predominantly help in defense against pathogens, parasites, and predators.

Research reports corroborate that the phenolic compounds possess antioxidants, anti-bacterial, anti-atherosclerotic, anti-cancer, anti-viral and anti-inflammatory activities [14,15,16]. Supplementing phenolics rich diet reduces the risk of cardiovascular diseases [17,18]. Flavonoids showed anti-allergic, anti-inflammatory, anticancer, antithrombotic, antimicrobial, antiviral, and hepato-protective properties owing to their ability in scavenging the free radicals effectively [2,18,19]. Terpenoids have been reported with antibiotic, antiseptic, anti-helminthic and insecticidal activities [18,20].

### 2.2. Characterization of G. latifolia Methanolic Fruit Extract

#### 2.2.1. FTIR Analysis

**Figure 1:**
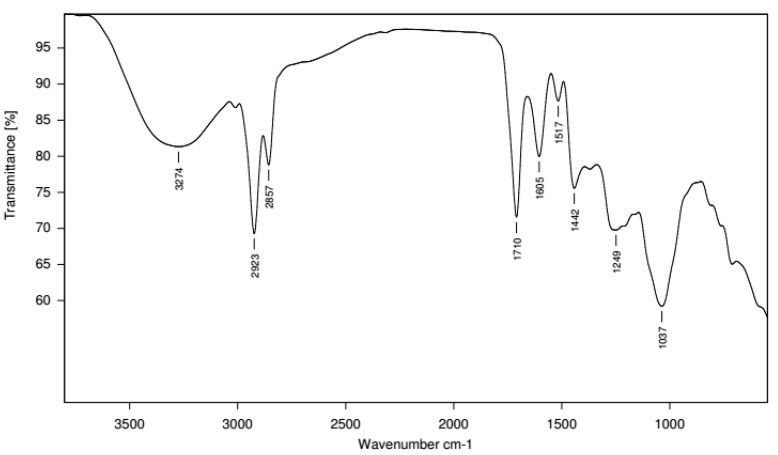
FTIR spectrum of methanolic fruit extract of *G. latifolia.*

The FTIR analysis was used to identify the functional groups of the phytoconstituents present in the extract on the basis of peak value in the infrared region (Figure 1) (Table S2). Methanolic fruit extract of *G. latifolia* showed the characteristic absorption bands at 3274 cm^−1^ (OH stretching), 2923 cm^−1^ (aromatic CH stretching), 2857cm^−1^ (for functional group), 1710 cm^−1^ (C=O stretching), 1605 cm^−1^ (C=C stretching), 1517 cm^−1^ (C=C stretching), 1442 cm^−1^ (C=C stretching), 1249 cm^−1^ (C=O stretching vibration), 1037 cm^−1^ (C-O-C stretching vibration), respectively [21]. The characteristic absorption band at 3274 cm^−1^ pertinent to OH stretching showed the presence of hydroxyl group, which is common in all phenolic compounds and implies the presence of phenolic compounds in methanolic extract of *G. latifolia*.

#### 2.2.2. LC–MS/MS Analysis

**Figure 2:**
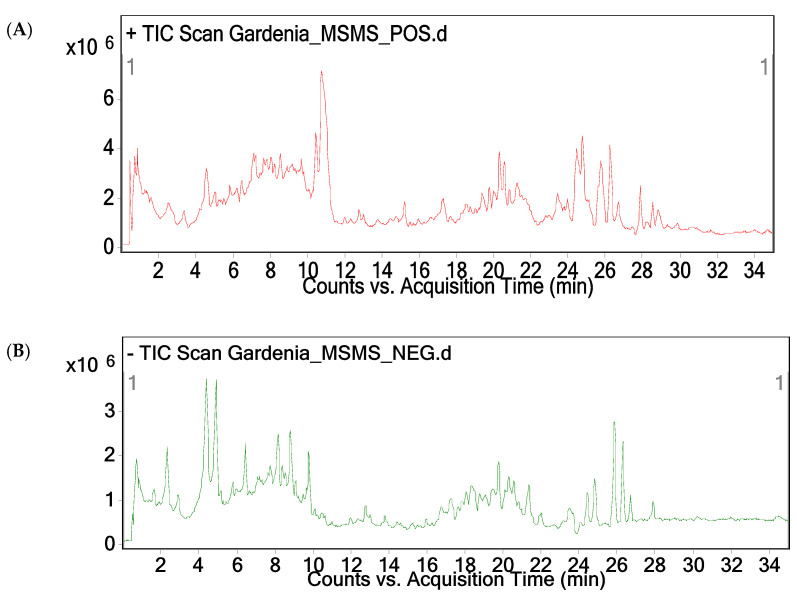
LC-MS/MS spectrum of methanolic fruit extract of *G. latifolia* (A) Chromatogram of fruit extract of *G. latifolia* on positive ionization mode. (B) Chromatogram of fruit extract of *G. latifolia* on negative ionization mode.

The LC-MS/MS analysis of the methanolic fruit extract of *G. latifolia* revealed the presence of 22 major compounds. Major peaks attributed in the chromatogram were subjected to both positive and negative modes using ESI-MS. Furthermore, the ion peaks along with MS fingerprint of compound was compared with other literature for identification of compounds. As per the LC-MS/MS analysis, 22 compounds consist of phenols, saponins, glycosides, alkaloids, flavonoids and terpenoids (Figure 2, Table 1). Some of the compounds such as 3,4-Di-O-caffeoylquinic acid, 6″-O-trans-feruloylgenipin gentiobioside, 10-(6-O-trans sinapoyl glucopyranosyl) gardendiol and 3-caffeoyl quinic acid (chlorogenic acid) showed hepato-protective and anti-viral activities and neuro-protective agent for Alzheimer’s disease, respectively [22,23,24].

Some other compounds like 3-caffeoylquinic acid (chlorogenic acid) and isoquercitrin were reported to be used in the treatment of osteoporosis and chemoprotective effects both in vitro and in vivo, against cancer, cardiovascular disorders, diabetes, oxidative stress and allergic reactions [25,26]. Scortechinones showed significant antibacterial activity against methicillin-resistant bacteria *Staphylococcus aureus* (MRSA) [27]. Secaubryenol is a pentacyclic triterpenoid and has been shown to exhibit a variety of anticancer activities against breast, prostate and cervical cancers [28]. Iridoids showed positive health effects on anti-inflammation, anti-depression, anti-diabetic properties and anti-thrombotic activities [29]. Quercetin-3-rutinoside (rutin), a natural flavonoid glycoside, showed antioxidant, hepatoprotective activities [30].

### 2.3. In vitro Antioxidant Activity of G. latifolia Methanolic Fruit Extract

#### 2.3.1. DPPH Free Radical Scavenging Assay

**Figure 3:**
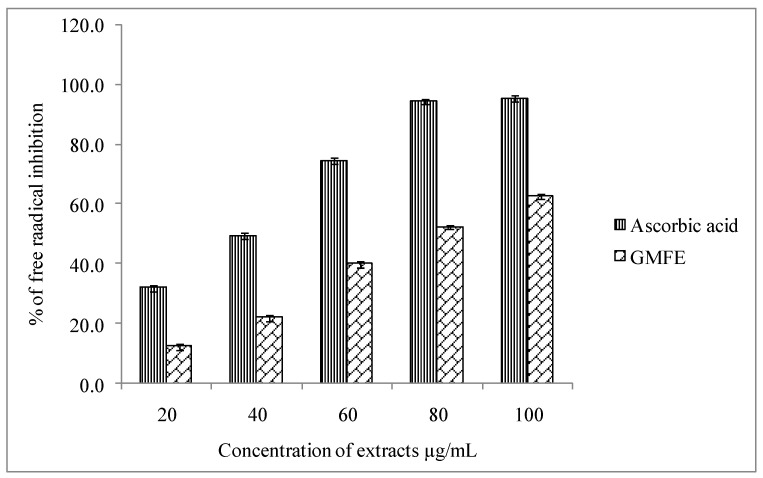
DPPH free radical scavenging assay of methanolic fruit extract of *G. latifolia*.

In the present study, *G. latifolia* methanolic fruit extract showed significant inhibition of DPPH free radicals. Dose response curve of DPPH radical scavenging activity of *G. latifolia* fruit extract in relation to ascorbic acid (standard) has been depicted in Figure 3. The IC50 value of *G. latifolia* methanolic fruit extract was 65.82 µg/mL, whereas IC50 value of ascorbic acid was 43.03 µg/mL. DPPH (a stable, nitrogen centered free radical) assay is one of the most widely used methods for screening antioxidant activity of plant extracts [31]. The main principle involved in DPPH assay is reduction of purple color methanolic DPPH solution to yellow color diphenyl-picryl hydrazine in the presence of hydrogen donating antioxidants. Decreasing absorbance indicates more antioxidant activity of the extract in terms of hydrogen donating capacity. Antioxidant property of *G. latifolia* methanolic extract may be attributed to the presence of polyphenol components, which can readily donate either hydrogen atoms or electrons to capture the free radicals.

Phenolic compounds play vital role in scavenging the free radicals. Several studies substantiate the positive correlation of quality of phenolics with the DPPH free radical scavenging effect [32,33]. Although methanolic fruit extract of *G. latifolia* was significantly less effective than ascorbic acid but showed stronger scavenging activity than those of the fruit extracts of *Gardenia volkensii* [34] and *Gardenia jasminoides* [35]. From this study, it is imperative that the methanolic fruit extract of *G. latifolia* has showed significant antioxidant activity owing to the presence of phenolic compounds.

#### 2.3.2. Differential Pulse Voltammetric Method

**Figure 4:**
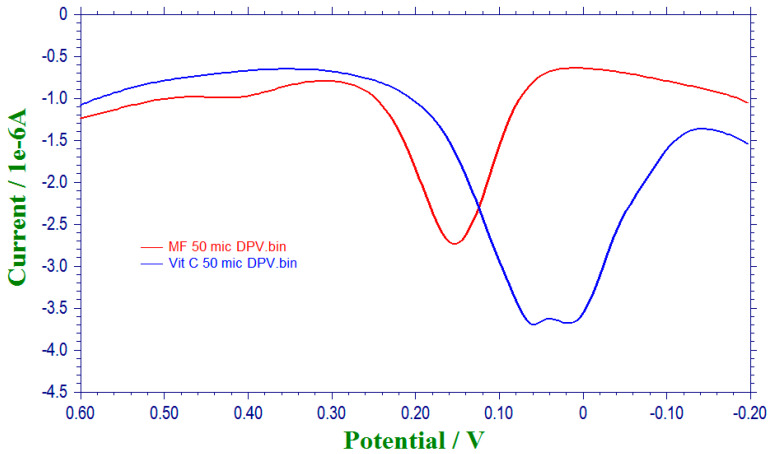
Differential pulse voltammograms of vitamin C (Vit-C) and methanolic fruit (MF).

Antioxidant potential of methanolic extract and standard ascorbic acid is shown in the Figure 4. The antioxidant potential of vitamin C is 0.060 V, whereas the methanolic extract of *G. latifolia* is 0.152 V. In cyclic voltammetry analysis, lower the oxidation potential higher the antioxidant activity of the compound [36]. Here the methanolic fruit extract showed higher oxidation potential (0.152 V) than the ascorbic acid (0.060), which indicates that the methanolic fruit extract is having lower antioxidant activity compared to ascorbic acid. Same results have been observed with the DPPH radical assay, which indicates that the method is efficient, reproducible, rapid and consume less time, solvent and energy.

#### 2.3.3. Antimicrobial Assay of *G. latifolia* Methanolic Fruit Extract

**Figure 5:**
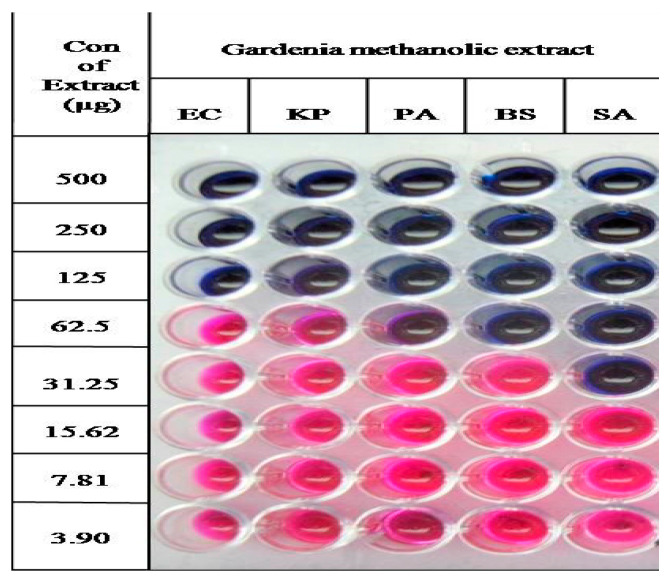
Minimum inhibitory concentrations of methanolic fruit extract of *G. latifolia* against Gram negative and positive bacteria.

MIC values of methanolic extract against Gram positive and negative bacteria have been depicted in Figure 5. The MIC values of *Escherichia coli*, *Klebsiella pneumoniae*, *Pseudomonas aeruginosa*, *Bacillus subtilis* and *Staphylococcus aureus* were 62.5 µg/µL, 62.5 µg/µL, 31.25 µg/µL, 31.25 µg/µL and 15.62 µg/µL, respectively. Resazurin is a non-toxic, non-fluorescent dye, which appears pink and fluorescent upon reduction to resorufin by oxidoreductases in the viable cells. This compound has been used as an indicator for the evaluation of cell growth, particularly in various cytotoxicity assays [37].

Methanolic extract showed significant inhibitory activity against methicillin resistant bacteria such as *Staphylococcus aureus*, which may be due to the presence of scortechinones. Similar findings were observed in *Garcinia scortechinii* by Sukpondma et al. [27]. Microtiter plates with pink color after 24 h of incubation in modified resazurin assay indicate the growth, while the microtiter plates with blue signifies no growth. This assay is helpful to screen the natural product bioassay both in vitro and in vivo and also cost-effective, rapid and easy to perform. Ansari et al. [6] have studied antioxidant and antidiabetic properties using the *G. latifolia* bark, but devoid of fruit extraction studies. Similarly, Sundar and Habibur [5] reported phytochemical compounds and antioxidants from bark, leaf and fruit. However, elucidation of phytochemical compounds of *G. latifolia* with LC-MS/MS has not reported. Hence, in the current study, phytochemical compounds pertinent to methanolic fruit extract of *G. latifolia* have been determined with LC-MS/MS, demonstrated antioxidant and antimicrobial activities with differential pulse voltammetry and resazurin tests. This study shows that the fruit extracts of *G. latifolia* can be used as medicinal extract and also illustrates the application of differential pulse voltammetry and resazurin tests for antioxidant and antimicrobial activities from fruit extracts.

## 3. Materials and Methods

### 3.1. Collection of Plant Material

Mature fruits of *Gardenia latifolia* Ait. were collected during the month of November, 2019 from Bhakarapeta (Latitude: 13°38′48.751″N and Longitude: 79°12′35.574″E) of Seshachalam Biosphere Reserve, Tirupati, A.P., India and authentication was done by Taxonomist, Department of Botany, Sri Venkateswara University, Tirupati. The shade dried and matured fruits were rinsed with running tap water followed by sterile distilled water to remove the dirt on the surface and cut into small pieces. They were then shade dried for 7 days at room temperature.

### 3.2. Preparation of Extracts

The shade dried fruits of *G. latifolia* were made into fine powder using electronic blender and kept in desiccator till further use. An amount of 20 g of fruit powder was extracted using 100 mL methanol at room temperature on magnetic stirrer for 72 h. Extract was filtered through Whatman No.1 filter paper, filtrate was collected and the solvent was evaporated by keeping on water bath at 60 °C. The concentrated extract was stored at 4 °C until further analysis.

### 3.3. Characterization of G. latifolia Methanolic Fruit Extract

#### 3.3.1. FTIR Analysis

FTIR spectrum of methanolic fruit extract was carried out on Alpha FT-IR spectrophotometer (Bruker, Germany) at a frequency of 4000–400 cm^−1^. About 1 mg of extract was used to record the spectrum.

#### 3.3.2. LC-MS/MS Analysis

LC-MS/MS analysis pertinent to methanolic fruit extract of *G. latifolia* was done using Agilent LC instrument (1200 infinity series, Santa Clara, California, USA). LC instrument is attached with time-of flight (TOF) mass spectrometer quadrupole (G6540B, Agilent Technologies) coupled with photodiode array detector (PDA) through electron spray ionization (ESI) interface using C18 column (4.6 mm × 250 mm, 5 µm). Mobile phase comprises of water: formic acid (100:0.1) (A) and acetonitrile (B). The gradient (linear) was set at 7–20 % B in 0–10 min, 20–30 % B in 10–13 min, 30 % B in 13–17 min, 30–7 % B in 17–18 min and 7 % in 18–25 min with a flow rate of 1 mL/min using 20 µL of sample injection volume. The column oven temperature was kept at 25 °C. Data acquisition and processing was performed using Mass Hunter Workstation software and the mass analyses were done by both positive and negative ion modes, respectively. Mass spectrometer has been programmed for three consecutive scans, where initial scan was done for full mass with a *m*/*z* 100–1000, thereafter finding most abundant ion in full mass (MS^2^) followed by most abundant ion in the full mass (MS^3^).

### 3.4. In vitro Antioxidant Activity of G. latifolia Methanolic Fruit Extract

#### 3.4.1. DPPH Free Radical Scavenging Assay

The DPPH free radical scavenging assay of methanolic extract was determined according to Chang et al. [38] with minor modifications. Stock solutions of methanolic fruit extract of *G. latifolia*, ascorbic acid and BHT were diluted at concentrations ranging from 20–100 μg/mL with an increment of 20 μg/mL. A volume of about 0.3 mL of standard/ sample was mixed with 2.7 mL of DPPH (0.1 mM) solution and the reaction mixture was incubated at room temperature in dark conditions for 30 min. Thereafter, the absorbance of mixture samples was measured against blank at 517 nm using double beam UV-Visible spectrophotometer (ELICO, Hyderabad, India). An amount of 3 mL of 0.1 mM DPPH solution was treated as control. Percentage (%) of free radical scavenging activity of methanolic fruit extract was calculated as per the formula given below.

: % inhibition = [(absorbance of control − absorbance of test sample)/absorbance of control] × 100

Where, Abs sample refers to the absorbance of plant extract + DPPH radical, Abs control denotes absorbance of Methanol +DPPH radical and RSA implies Radical Scavenging Activity.

The IC50 value, which is defined as the amount of sample concentration required to scavenge 50% DPPH free radicals was deduced from the plot of inhibition (%) against the sample extract concentration. All experiments were carried out in triplicate and the average of the results was reported. BHT and ascorbic acid were used as standards.

#### 3.4.2. Differential Pulse Voltammetric Method

Antioxidant potential of methanolic fruit extract of *G. latifolia* was determined as per the Amidi et al. [39] method with minor modifications. Differential voltammetric measurements were performed in conventional three electrode system pertinent to a single compartment cell. The three electrode system comprises of saturated calomel reference electrode (SCE), glassy carbon working electrode (GCE) and platinum wire auxiliary electrode. Before measurement of each sample, the working electrode was polished with alumina using polishing cloth and the electrochemical measurements were recorded on Metrohm VA 746 polarograph. The methanolic extract and standard (ascorbic acid) were prepared with a concentration of 5 mg/mL. In DPV experiment, 50 µL of each methanolic and ascorbic acid solution (5 mg/mL) were added to 2950 µL of 0.2 M phosphate buffer having pH 7.0 at 70 mV pulse amplitude and 5 mV/s. The voltammograms were recorded between −0.2 V to 0.6 V.

### 3.5. Determination of Antimicrobial Potential of Methanolic Fruit Using Resazurin Microtiter Plate Assay

#### 3.5.1. Bacterial Cultures and Culture Media

Bacterial strains such as Gram-positive *Staphylococcus aureus* (ATCC 25923), *Bacillus subtilis* (ATCC6633) and Gram-negative *Escherichia coli* (ATCC 25922), *Pseudomonas aeruginosa* (ATCC 27853), *Klebsiella pneumoniae* (ATCC 700603) were obtained from NCIM Resource Centre, Pune, India and were used in the present study. Bacterial cultures were cultivated on Mueller–Hinton broth (MHB) (HiMedia, Mumbai, India) and incubated as per the protocol supplied by NCIM resource centre, Pune, India. Bacterial cultures were suspended in 10 mL of physiological saline solution until the optical density readings reached to 0.5 McFarland standard. Further, to calculate minimum inhibitory concentration (MIC) of bacterial solutions 5 × 10^5^ colony-forming units (cfu) mL were used.

#### 3.5.2. Preparation of Microtiter Plates

The MIC value of methanolic fruit extract was determined using sterile 96-well microtiter plate with resazurin as an indicator for cell growth [11]. A 96 well microtiter plate (Tarsons, Kolkata, India) was prepared by transferring 100 μL of Mueller–Hinton broth under laminar air flow chamber. A volume of 100 μL of methanolic extract (10 mg/mL) in 10% (*v*/*v*) DMSO was added into the first row of the plate and serial dilutions were performed. To each well, initially 10 μL of resazurin solution was added followed by 10 μL of bacterial inoculum (5 × 10^6^ cfu/mL). Thereafter, cling film was loosely wrapped to the microtiter plate to avoid bacterial culture dehydration and the plates were incubated at 37 °C for 24 h in an incubator. Colour change in the wells was observed visually, where change from purple to pink colour or colourless was scored as positive. MIC value was determined by taking the lowest concentration of the extract in which the colour change occurred. All the experiments were carried out in triplicates and the average values were measured for the MIC of the test material.

## 4. Conclusions

Phytochemical compounds of *G. latifolia* fruits showed several secondary metabolites such as saponins, alkaloids, glycosides, phenols, terpenoids and flavonoids having various putative functions. Antioxidant activity of methanolic fruit extract showed that it has huge potential to be used in food industry. Unlike conventional tests, this study has evaluated the antioxidant activity from the sample using differential pulse voltammetric method and showed reproducible results with DPPH assay. Besides, resazurin tests also showed highly reproducible results, which can be replaced with the conventional tests as they are rapid, reproducible, cost-effective and consume less time, energy and solvents. This study further emphasizes to use the *G. latifolia* fruit extract for the development of phyto-therapeutics owing to the presence of diversified biologically active compounds.

## Figures and Tables

**Table 1:** Compounds identified in methanolic fruit extract of *G. latifolia* by LC-MS/MS.

S. No.	Molecular Formula	*m*/*z*	RT	Mass	Name of The Compound	Mode +/−
1.	C_11_ H_12_ O_5_	225.0752	3.294	224.0679	Sinapinic acid	+
2.	C_16_ H_12_ O_6_	301.0709	11.969	300.0636	4′Hydroxywogonin	+
3.	C_16_ H_18_ O_9_	355.1029	2.281	354.0956	3-caffeoylquinic acid (Chlorogenic acid)	+
4.	C_18_ H_22_ O_10_	397.1154	5.302	398.1228	3-O-sinapoylquinic acid	−
5.	C_19_ H_22_ O_5_	331.1553	7.731	330.1479	5-Deoxystrigol	+
6.	C_20_ H_20_ O_8_	389.1234	16.589	388.1161	5 Hydroxy6,7,3′, 4′,5′pentamethoxyflavone	+
7.	C_20_ H_20_ O_9_	403.1044	15.14	404.1118	5,3′Dihydroxy3,6,7,4′, 5′pentamethoxy flavone	−
8.	C_20_ H_32_ O_2_	305.2476	22.752	304.2403	2-Ketoepimanool	+
9.	C_21_ H_20_ O_12_	465.1031	7.685	464.0957	Isoquercitrin	−
10.	C_25_ H_24_ O_12_	517.1344	8.177	516.1269	3,4-Di-O-caffeoylquinic acid	+
11.	C_26_ H_30_ O_13_	549.1627	8.502	550.1699	2′-O-trans-feruloylgardoside (Iridoid glycosides)	−
12.	C_26_ H_30_ O_14_	565.159	7.534	566.1661	6′-O-[(E)-caffeoyl]-deacetylasperulosidic acid methyl ester (Iridoid glycosides)	−
13.	C_27_ H_28_ O_13_	561.1609	9.684	560.1535	4-O-sinapoyl-5-O-caffeoylquinic acid	+
14.	C_27_ H_30_ O_16_	609.1496	7.451	610.1569	Quercetin-3-rutinoside (Rutin)	+
15.	C_27_ H_34_ O_20_	677.1545	10.708	678.1617	10-(6-O-trans-sinapoylglucopyranosyl) gardendiol	−
16.	C_30_ H_48_ O_3_	455.354	23.505	456.3613	Betulinic acid	−
17.	C_33_ H_42_ O_18_	725.2302	9.186	726.2369	6″-O-trans-feruloylgenipin gentiobioside (Iridoid glycosides)	−
18.	C_34_ H_40_ O_10_	609.271	24.561	608.2637	Scortechinone C	+
19.	C_34_ H_40_ O_9_	593.2769	25.688	592.2695	Scortechinone B	+
20.	C_35_ H_42_ O_9_	607.2923	27.925	606.285	Scortechinone G	+
21.	C_35_ H_44_ O_10_	625.3027	25.124	624.2953	Scortechinone I	+
22.	C_6_ H_8_ O_6_	177.041	5.731	176.0337	Ascorbic acid	+

## Supplementary Materials

The following are available online at https://www.mdpi.com/2223-7747/10/3/545/s1, Table S1. Preliminary phytochemical analysis of G.latifolia fruit extracts. Table S2. FTIR spectral peak values and functional groups of the methanolic fruit extract of G.latifolia.
