# Supplementary material for: Phytochemical Profiling of Methanolic Fruit Extract of Gardenia latifolia Ait. by LC-MS/MS Analysis and Evaluation of Its Antioxidant and Antimicrobial Activity
Source: Plants (Basel). 2021 Mar 13;10(3):545. doi: 10.3390/plants10030545 (PMC7998288; doi:10.3390/plants10030545)
Supplement: Supplementary file 1 [file plants-10-00545-s001.pdf]

Supplementary Data

Table S1. Preliminary phytochemical analysis of *G.latifolia* fruit extracts.

**Solvent extracts**	**Phytochemicals**					
	**Alkaloids**	**Phenols**	**Flavonoids**	**Saponins**	**Glycosides**	**Terpenoids**
Hexane	-	-	-	-	-	-
Chloroform	-	+	+	-	-	-
Ethyl acetate	-	+	+	-	+	+
Methanol	+	++	+	+	+	+++

"+" indicates the presence of constituents "-" indicates the absence of constituents

Table S2. FTIR spectral peak values and functional groups of the methanolic fruit extract of *G.latifolia*

**Peak Values (cm^{-1})**	**Functional Groups**
3274	OH stretching
2923	aromatic CH stretching
2857	aromatic CH stretching
1710	C=O stretching
1605	C=C stretching
1517	C=C stretching
1442	C=C stretching
1249	C-O stretching vibration
1037	C-O-C stretching vibration
